# Non-disclosure of tuberculosis diagnosis by patients to their household members in south western Uganda

**DOI:** 10.1371/journal.pone.0216689

**Published:** 2020-01-24

**Authors:** Miria Nyangoma, Francis Bajunirwe, Daniel Atwine

**Affiliations:** 1 Department of Information Technology, Mbarara University of Science and Technology, Mbarara, Uganda; 2 Department of Community Health, Mbarara University of Science and Technology, Mbarara, Uganda; 3 Department of Clinical Research, Epicentre, Mbarara, Mbarara, Uganda; University of Botswana Faculty of Medicine, BOTSWANA

## Abstract

**Background:**

Tuberculosis (TB) non-disclosure by adult patients to all household members is a setback to TB control efforts. It reduces the likelihood that household contacts will seek early TB screening, initiation on preventive or curative treatment, but also hinders the implementation of infection controls and home-based directly observed treatment. Therefore, the purpose of this study was to determine the level of TB non-disclosure, its predictors and the effects of disclosure among adult TB patients in Uganda.

**Methods:**

We conducted a cross-sectional study at a large regional referral hospital in Mbarara, south-western Uganda. Questionnaires were administered to collect patients’ sociodemographic and their TB disclosure data. Non-disclosure was considered if a patient did not reveal their TB diagnosis to all household members within 2 weeks post-treatment initiation. Univariate and multivariate logistic regression models were fitted for predictors of non-disclosure.

**Results:**

We enrolled 62 patients, 74% males, mean age of 32 years, and median of five people per household. Non-disclosure rate was 30.6%. Post-disclosure experiences were positive in 98.3% of patients, while negative experiences suggestive of severe stigma occurred in 12.3% of patients. Being female (OR 6.5, 95% CI: 1.4–29.3) and belonging to Muslim faith (OR 12.4, 95% CI: 1.42–109.1) were significantly associated with TB non-disclosure to household members.

**Conclusions:**

There is a high rate of TB non-disclosure to all household members by adult patients in rural Uganda, particularly among women and muslim patients. Interventions enhancing TB disclosure at household level while minimizing negative effects of stigma should be developed and prioritized.

## Background

Tuberculosis (TB) remains one of the ten causes of death worldwide and the leading cause from a single infectious agent, ranking above HIV/AIDS [1]. The World Health Organization (WHO) estimated that 10 million people fell ill with TB in 2018 and that treatment success rate among new cases remained at only 85% by 2017 globally [1]. Uganda is among the top 30 TB-HIV high burden countries [1], with an overall TB incidence of 200 per 100,000 people and treatment success rate of 69% by 2018 [1–2].

In addition to the HIV epidemic, uncontrolled transmission is fueling the global tuberculosis epidemic [3]. All indoor environments especially homes and congregate settings are potential sites of transmission since air dilution is limited and occupants are crowded [4–5]. Adherence to effective treatment not only ensures a faster reduction in risk of TB transmission from adult patients to their household members [6], but also limits the emergence of drug resistance. WHO recommends both social and psychological support as supplements to community- or home-based directly observed treatment [7]. This ensures several successful outcomes namely: completion of treatment, prevention of relapse, death from active TB or its late effects, reduction in transmission to others [8], and prevention of the development and transmission of drug resistance [9–10]. Household members are well positioned to provide such needed social and psychological support so as to ensure successful treatment outcomes and infection control [11], but only if adequately engaged in care of their TB patient, a step that is dependent on patients’ disclosure of their TB diagnosis.

Disclosure of TB diagnosis to household members could influence patient adherence [12], but may also promote early initiation of appropriate infection control measures, and access to diagnostic, curative and preventive TB services by their household contacts. However, TB disclosure may be hindered due to TB-related stigma, the association between TB and Human immunodeficiency virus (HIV) infection, perceived incurability of TB, and myths about TB etiology [13]. Negative consequences of disclosure like patient isolation, neglect, withdrawal of social support, and divorce have been previously reported [14].

There is a paucity of information on the frequency of disclosure of TB disease status at household level and consequences of disclosure among patients within TB-HIV high-burden countries. This information is useful in designing contextualized interventions to promote disclosure, as part of the efforts to achieve the 2035 targets of the End-TB strategy [15]. Overall, knowledge on TB disclosure dynamics at household level, could guide TB control interventions. Therefore, we conducted a study at a large regional referral hospital in south-western Uganda, aimed at measuring the level of TB non-disclosure to household members by adult patients, the associated factors and patients’ experiences post- disclosure.

## Methods

### Design and setting

We conducted a cross sectional study among adult patients started on antituberculosis treatment for pulmonary TB at the TB clinic at Mbarara Regional Referral Hospital. The hospital is a large tertiary health facility in south-western Uganda with a catchment area of nearly 2 million people. Data were collected at the clinic between May and July 2017. In agreement with the National TB Control Program guidelines, each patient is required to identify someone close to them prior to initiation of TB treatment, to serve as a treatment supporter where possible. Trained nurses then counsel patients on TB disease, treatment adherence and infection control before administering treatment. Patients return for drug refills every 2 weeks during the first 2 months of treatment (intensive phase), and thereafter, monthly for the next 4 months (continuation phase).

### Study population

Patients were eligible for enrollment if they were 18 years or older, were either new or retreatment cases with bacteriologically confirmed or unconfirmed pulmonary TB, completed at least 2 weeks of TB treatment, living with at least one household member, and provided written consent. The unit was considered to be a household if there was more than one person living in the same dwelling; sharing meals and living accommodation.

Patients were excluded if they were too ill, or had psychiatric disorders deterring them from responding to interview questions. The informed consent process was administered by trained research assistant study nurses who were not participating in patient care within the TB clinic.

### Study procedures

We administered a structured questionnaire with both closed and open questions in English or translated into the local language of Runyankole based on the patient’s preference (S1 Questionnaire and S2 Questionnaire). We collected data on socio-demographic characteristics, household, patients’ behavioral factors such as alcohol intake, smoking, medical history, and patients’ TB disclosure experiences. We focused at assessing disclosure that occurs early during treatment and we defined this as TB disclosure to household members within the first 2 weeks following treatment initiation. The choice of the first 2 weeks was premised on being the estimated time for a patient on appropriate TB treatment to become non-infectious [16], and therefore, could serve as the best time to optimize adherence support but also infection control measures so as to minimize TB transmission at household level.

In addition, post-disclosure experiences were elicited by asking patients an open-ended question “Can you tell us what your experience was two weeks after disclosing your TB status to your household members?” Based on the response, the research assistant checked “Yes” or “No” on a list of responses in the listing or would fill in the space provided for “specifying other experiences” that might not have been listed. The open question allowed the patient to respond freely without bias.

The predefined positive post-disclosure experiences were: 1) encouragement from family members, 2) support in taking their medications, 3) support in feeding, 4) financial support to attend clinic days, 5) Other positive experiences, in which case, the researcher would specify the additional positive experiences reported by the patient that were outside the predefined list. The negative post-disclosure experiences of patients elicited by the researcher focused on demonstrating any effects on the patient that is due to potential TB-related stigma among household members. These included experiences of 1) criticism or blame, 2) isolation by household members, 3) withdrawal of support by household, 4) marital separation, and 5) other negative experiences, in which case, the researcher would specify the additional negative experiences reported by the patient that are outside the predefined list.

Patients were then asked to respond with a “Yes” or “No” on whether they perceived that the above positive or negative post-disclosure experiences had impacted on: their treatment intake and adherence to scheduled TB clinic visits, but also on some of their psychosocial aspects, that is; self-esteem, hope of recovery, work, household harmony, and marital relationships in the case of those who were married or co-habiting. Also, patients were asked on whether they would agree to the training of their treatment supporters by healthcare workers so as to support in patients’ TB disclosure to household members.

The primary dependent variable for this study was “disclosure”, which was derived from a question on whether they had “disclosed their current TB status to their household members”, with three responses namely “yes, all = 1”, “yes, some = 2” and “no = 3”. A patient was considered to have complete disclosure if he or she responded with “yes, all”, meaning that they had revealed their TB diagnosis to all their household members. A patient was considered to have partial disclosure if he or she responded with “yes, some”, meaning that he or she had revealed their TB diagnosis to only a selected number of their household members and left out others.

For the purpose of the analysis of study outcomes, we generated a binary dependent variable coded 0 = complete disclosure, and 1 = partial or non-disclosure. The patients were considered to have disclosed only if they had complete disclosure, and everyone else was considered as “no disclosure’. The decision was made because both total non-disclosure and partial disclosure positions household members at risk of TB infection, given that TB is an infectious disease. The independent variables included all patients’ characteristics of socio-demographics, household characteristics, patients’ behavioral factors alcohol intake and smoking and medical characteristics.

### Sample size

A sample size of 62 patients was calculated using a formula for single population proportion with correction for finite population [17]. In the calculation, sample size n = N*X / (X + N– 1), where, X = Z_α/2_^2^ *p*(1-p) / MOE^2^, and Z_α/2_ is the critical value of the Normal distribution at α/2 for α of 0.05, MOE is the margin of error considered at 5%, p is the proportion of non-disclosure which was taken as 50%, an arbitrary value given the lack of comparable data, and N is the population size, estimated at 70 new patients with TB undergoing treatment at the clinic for two weeks to two month period. To cater for attrition, an additional 5% was added to the sample size.

### Data analysis

Data from completed questionnaires were entered into a database designed using Epi Info^™^ software (V7.2, 1600 Clifton Road Atlanta, GA 30329–4027 USA) and analysis was performed with Stata software (v.13, College Station, Texas, USA). Descriptive statistics were generated for participants’ characteristics. The proportion of patients reporting not to have disclosed their TB status (that is, partially or completely) to household members was calculated out of all study participants and presented as a percentage. Percentages of TB patients with positive or negative post-disclosure experiences, and those reporting different aspects of their lives impacted by these post-disclosure experiences at household level were calculated as proportions. The dependent variable was a binary variable of TB status non-disclosure to household members coded 0 = No and 1 = Yes.

All patient factors were used as independent variables in this analysis. In Univariate analysis, based on both Chi-square test and Logistic regression, analysis comparing each independent variable with non-disclosure was performed. Unadjusted odds ratios with their corresponding 95% CI were reported. A significance level of 5% was used. All factors with p-value <0.1 in univariate analysis and those with biological plausibility (e.g. age categories) were considered in the multivariate analysis which was performed to control potential confounding. Assumptions for use of multiple logistic regression, e.g. the absence of multicollinearity among the independent variables, were checked. A manual back-ward stepwise selection method was used in fitting the final multivariate analysis model. In this method, we excluded variables that lost their meaningful association with TB non-disclosure after controlling for the effect of other variables in the model. The goodness-of-fit test was performed on the final model to assess its quality. The factors in the final multivariate model were reported together with their adjusted odds ratios and 95% confidence intervals. A variable was considered significant in this analysis if it had a p<0.05.Percentages were calculated to describe the patients’ perception on training treatment supporters so as to enhance their role in supporting patients’ disclosure at household level.

### Human subjects

The study was approved by the Mbarara University Faculty of Medicine Research Committee, and Mbarara University of Science and Technology Research Ethics Committee. Study approval no: 16/02-17. Written informed consent was received from all study participants prior to enrollment in the study. We also used separate research assistants (nurses) who were not involved in the routine care of these TB patients to perform the interviews. The patients’ treatment was a priority to research and they were only interviewed after they had received their routine care, so as to prevent coercion and therapeutic misconception. No incentives were given for patients’ participation in the study. All patients’ information was kept confidential.

### Results

We enrolled 62 patients, predominantly males (74%), mean age of 32 years, with half having at least secondary education. Majority (80%) of patients’ households had atleast four people. About 95% of the patients had the current episode as their first TB episode, 82% had a treatment supporter, 49% were HIV infected, and two-thirds had been on TB treatment for at least 8 weeks at the time of interview. The details of the sociodemographic characteristics are shown in Table 1.

**Table 1:** Participants’ characteristics.

Characteristics	N	n (%)
**Age in years, mean (SD)**	62	32.3 (8.6)
**Age categories, n (%)**	62	
<25		12 (19.4)
25–34		27 (43.6)
35–59		23 (37.1)
**Gender, Males, n (%)**	62	46 (74.2)
**Residence, Urban, n (%)**	62	36 (58.6)
**Education Level, n (%)**	62	
None		8 (13.0)
Primary		23 (37.1)
Secondary		24 (38.7)
Tertiary		7 (11.3)
**Marital status, single, n (%)**	61	34 (55.7)
**Occupation, n (%)**	62	
Un employed		6 (9.7)
Business		23 (37.1)
Peasant farmer		13 (21.1)
Professional		15 (24.2)
**Family, n (%)**	62	
Extended		15 (24.2)
Nuclear		42 (67.7)
Single parent		4 (6.5)
**Religion**	62	
Christian		55 (88.7)
Moslem		7 (11.3)
**Household members, ≥4, n (%)**	61	49 (80.3)
**First TB episode, n (%)**	62	59 (95.0)
**Treatment duration at the time of data collection**	62	
**Median treatment duration (IQR)**		12.4 (6.4–21.3)
Treatment duration above 8 weeks, n (%)*		40 (65.6)
**Treatment supporter, n (%)**	62	51 (82.3)
**Monthly income in Ug.sh, median (IQR)**	47	100,000 (50,000–200,000)
**HIV positive, n (%)**	62	30 (48.4)
**Has dependants, n (%)**	62	39 (62.9)
**Patient is household head, n (%)**	62	31 (50.0)
**Alcohol history prior to TB, n (%)**	62	39 (62.9)
**Currently Smoking, n (%)**	62	16 (25.8)
**Ever experienced suicidal thoughts**	62	5 (8.1%)

*1 missing response; IQR, interquartile range; SD, standard deviation; TB, tuberculosis; Ug.sh, Ugandan shillings

### TB non-disclosure to household members

Overall, of the 62 patients, 19 (30.6%) had either not disclosed to some or all the members of their household. Significantly higher TB non-disclosure rates were noted among patients who were; female (62.5%, p = 0.001), single (44.1%, p = 0.005), and moslems (71.4%, p = 0.013), as compared to their counterparts. No significant differences in non-disclosure rates were observed across patients’ age, occupation, education and residence type, p>0.05. The detail of these results are shown in Table 2.

**Table 2:** TB Non-disclosure rates across patients’ sociodemographic characteristics.

Non-disclosure proportion	n/N	% [95% CI]	p value
**Overall proportion**	19/62	30.7 [20.2–43.5]	
**Gender-specific proportion**			0.001
Male	9/46	19.6 [10.2–34.1]	
Female	10/16	62.5 [34.8–83.9]	
**Age-specific proportion**			0.492
<25	4/12	33.3 [10.9–67.1]	
25–34	10/27	37.0 [20.3–57.5]	
35–59	5/23	21.7 [8.7–44.8]	
**Marital status**			0.005
Single	15/34	44.1 [27.9–61.7]	
Married	3/27	11.1 [3.3–31.1]	
**Religion**			0.013
Christian	14/55	25.5 [15.4–39.0]	
Moslem	5/7	71.4 [21.5–95.8]	

CI, confidence interval

Overall, the median duration on TB treatment for patients that had not disclosed to household members by time of interview was 13.3 (IQR 2.0–13.2) weeks.

Of the five patients that had not disclosed to any one, four had been on treatment for more than 4 weeks at the time of interview.

### Motivation for TB disclosure

Of the 57 patients with either complete or partial disclosure, 50 responded to the open-ended question on what had motivated their TB disclosure to household members. The responses were, 1) The desire to receive support/care from household members (40%); 2) need to avoid transmitting the disease (30%); 3) Need for support to collect and remind them to take their medicines (16%); and then, 4) the need to inform their family what they were suffering from (14%).

### Factors associated with TB non-disclosure

In univariate analysis, the factors significantly associated with adult TB non-disclosure to household members are; female gender, being a Moslem, being single or unmarried and having dependents, as shown in Table 3.

**Table 3:** Results of univariate analysis for factors associated with non-disclosure of adult TB status to household members.

Variable	No disclosure, n (%)	Disclosed, n (%)	Un adjusted OR (95% CI)	p
Age categories in years				0.4826
<25	8 (66.7)	4 (33.3)	Ref	
25–34	17 (63.0)	10 (37.0)	1.2 [0.28–4.93]	
35–59	18 (78.3)	5 (21.7)	0.6 [0.12–2.63]	
**Gender**				0.0018
Male	37 (80.4)	9 (19.6)	Ref	
Female	6 (37.5)	10 (62.5)	6.9 [1.97–23.84]	
**Marital status,**				0.0035
Single	19 (55.9)	15 (44.1)	6.3 [1.59–25.05]	
Married/cohabiting	24 (88.9)	3 (11.1)	Ref	
**Religion**				0.0867
Christian	41 (74.6)	14 (25.5)	Ref	
Moslem	2 (28.6)	5 (71.4)	7.3 [1.27–42.07]	
**Education level**				0.9514
None	6 (75.0)	2 (25.0)	Ref	
Primary	15 (65.2)	8 (34.8)	1.6 [0.26–9.83]	
Secondary	17 (70.8)	7 (29.2)	1.2 [0.20–7.67]	
Tertiary	5 (71.4)	2 (28.6)	1.2 [0.12–11.87]	
**No of household members**				0.4494
1–3	10 (83.3)	2 (16.7)	Ref	
4–5	13 (65.0)	7 (35.0)	2.7 [0.48–15.88]	
>5	19 (65.5)	10 (34.5)	2.6 [0.48–14.41]	
**Have dependents**	31 (79.5)	8 (20.5)	0.3 [0.09–0.87]	0.0255
**Not a household head**	18 (58.1)	13 (41.9)	0.3 [0.11–1.04]	0.0517
**HIV positive**	18 (60.0)	12 (40.0)	2.4 [0.78–7.24]	0.1204
**Treatment supporter**	36 (70.6)	15 (29.4)	0.7 [0.19–2.86]	0.6541

OR, odds ratio; CI, confidence interval; HIV, human immunodeficiency virus; Ref, reference category

However in multivariate analysis, the only factors with independent association with TB non-disclosure to household members were; female gender and being a Moslem, after controlling for age of patients at multivariate analysis. The odds of non-disclosure of TB status to household members were 6.5 times higher in females as compared to males (OR 6.5, [95%CI: 1.42–21.2], p = 0.016). Moslem patients had 12.4 times higher odds of non-disclosure of their TB status to household members as compared to Catholic patients (OR 12.4, [95% CI: 1.42–109.05], p = 0.023). Patients who were not married (single) had 4.6 times higher odds of non-disclosure of TB status to household members as compared to patients who were married, with a tendency towards significance (OR 4.6, [95% CI: 0.98–21.22], p = 0.053). See details in Table 4.

**Table 4:** Results of multivariate analysis for factors independently associated with non-disclosure of adult TB status to household members.

Variable	Multivariate analysis	
	Adjusted OR [95%CI]	p value
**Age categories in years**		
<25	Ref	
25–34	4.1 [0.50–33.08]	0.192
35–59	1.6 [0.19–14∙02]	0.660
**Gender**		0.016
Male	Ref	
Female	6∙5 [1.42–29.28]	
**Marital status,**		0.053
Single	4.6 [0.98–21.22]	
Married/cohabiting	Ref	
**Religion**		0.023
Christian	Ref	
Moslem	12.4 [1.42–109.05]	

OR, odds ratio; CI, confidence interval; HIV, human immunodeficiency virus; Ref: reference category

### TB patients’ positive post-disclosure experiences

**Fig 1:**
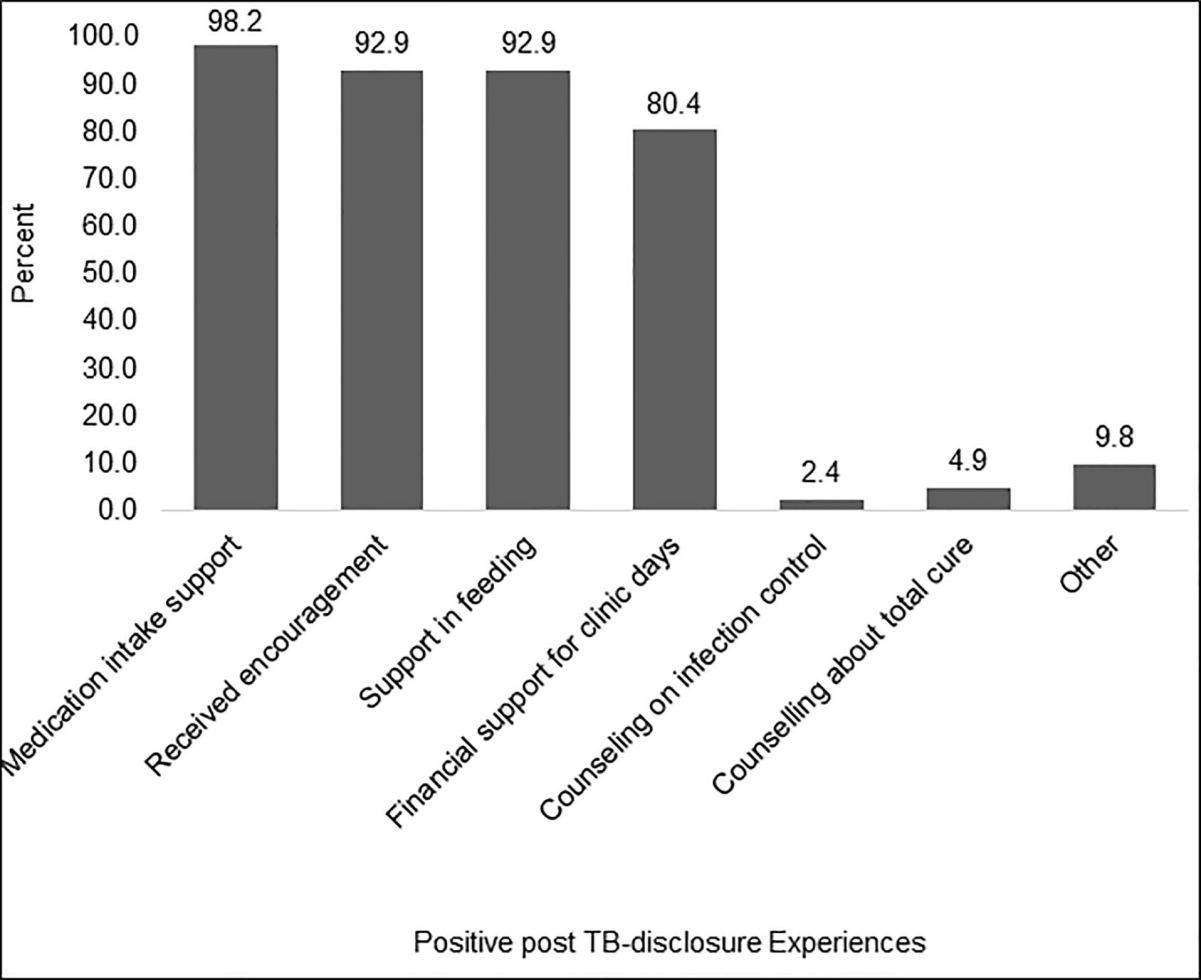
Positive post-disclosure experiences in adult TB patients, N = 57.

Of the 57 patients with either complete or partial disclosure, 56 (98.3%) reported positive experiences post-disclosure. These were in form of support from household members in areas of: medication intake; encouragement; in feeding; and financial support to enable attendance of clinic days. The areas with the least reported support offered by household members were; infection control (2.4%) and counselling about TB as a curable disease (4.9%) (Fig 1).

**Fig 2:**
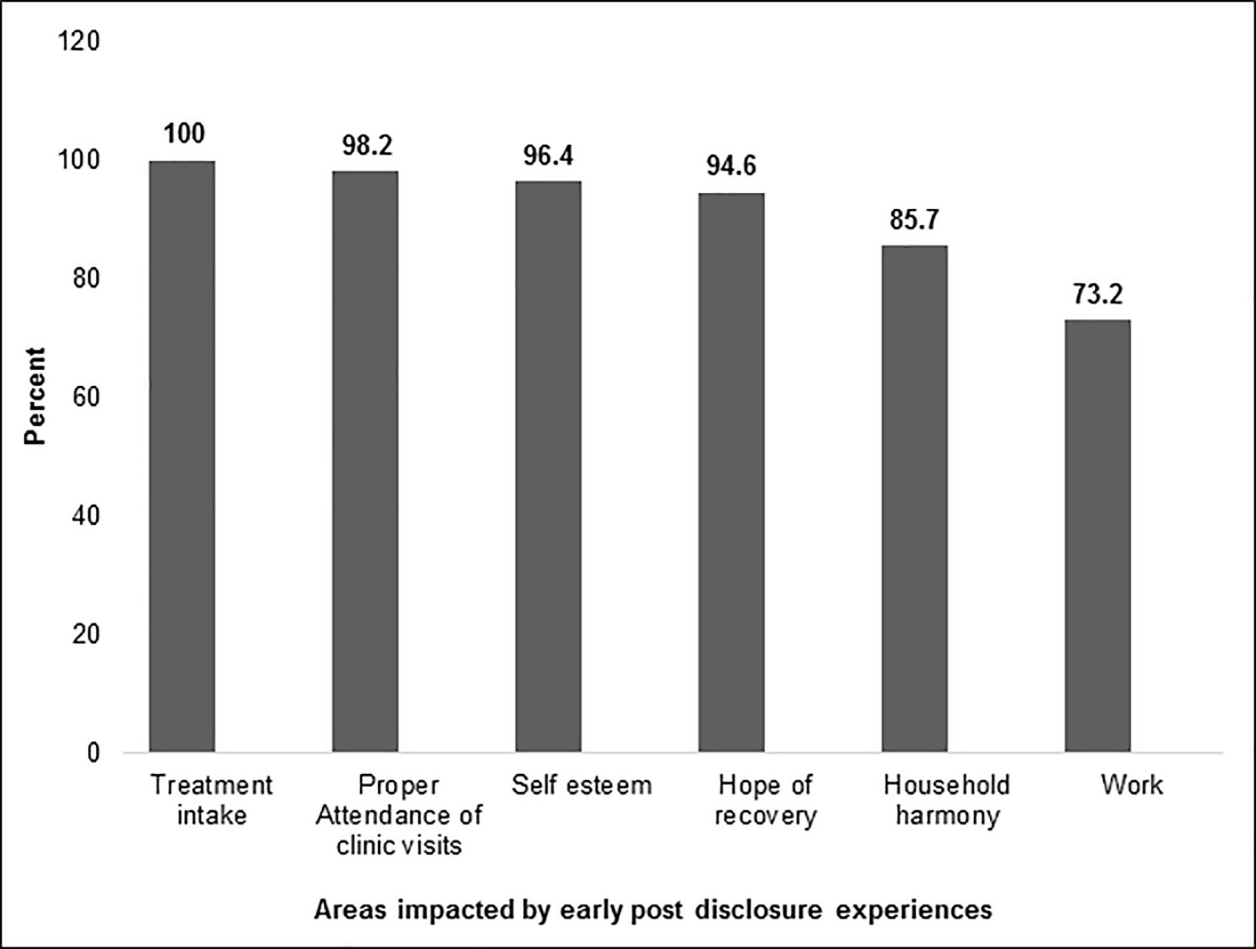
Areas of TB patients that are impacted by early positive post-disclosure experiences, N = 56.

Majority of patients felt that these positive experiences post disclosure, had impacted greatly on their: treatment intake, attendance of clinic visits, self-esteem, hope of recovery, household harmony and work (Fig 2).

### TB patients’ negative post-disclosure experiences

Of the 57 patients with either complete or partial disclosure, 7 (12.3%) had experienced atleast one negative experience following disclosure. The most common negative post-disclosure experiences among them were: marital separation (n = 3), blame and negative criticism (n = 2) and isolation (n = 2) All these 7 patients also reported to have had at least one positive post-disclosure experience.

### Perceptions towards enhancing treatment supporter role in ensuring complete patient disclosure

Of the 62 study participants, 38 (61.3%) expressed agreement to the involvement of a treatment supporter to assist in patients’ TB disclosure to household members through training and provision of suitable TB information aides (like charts) by healthcare workers.

## Discussion

Our study is the first, to the best of our knowledge, to explore the dynamics of TB disclosure at a household level in Uganda. Disclosure of TB status is a key strategy in promoting engagement of household members in TB control and efforts to achieve the targets of the End TB strategy [15]. We report a high rate of non-disclosure to household members by adult TB patients (30.6%). The need to attract care was the main motivational factor for patients to disclose to household members, besides other factors like: the desire to avoid transmitting the disease to them and attracting support in picking or reminding them to take their medicines.

TB disclosure to household members yielded positive benefits almost to all patients, especially with regard to treatment adherence and psychosocial support, but with little gains on counselling pertaining to infection control and hope of complete cure from TB. Negative experiences occurred to about one out of every eight TB patients following disclosure to household members. Being female or a Muslim was significantly associated with non-disclosure of TB diagnosis to household members. A potential intervention based on training and provision of suitable TB information aides to treatment supporters by healthcare workers, so as to enhance their confidence in supporting the TB patient in the process of disclosure to household members so as, was acceptable by two-thirds of TB patients.

The observed high TB non-disclosure rate among adult patients to their household members, is of great public health concern, and can result in uncontrolled TB transmission at household level. Some of the patient factors and their household characteristics in our study seem to be in alignment with this concern, and indicative of a conducive environment for continued TB exposure and potential transmission. The point in case is the fact that; 1) majority (80%) of TB patients were staying with at least 4 household members, who would therefore qualify as TB contacts and at-risk of TB infection; 2) most of the patients (95%) were newly diagnosed with TB, and though not explored in this study, but could perhaps have had insufficient experience and knowledge on TB infection control measures so as to be able to minimize transmission to their household members; and 3) as part of the study eligibility criteria, all patients had been at least 2 weeks on treatment, and we noted a median time of 13.3 weeks since treatment initiation among patients reporting non-disclosure to household members. This suggests a prolonged period of potential uncontrolled TB transmission to household members. Such non-disclosure deprives the patient with TB of the care and support they would have received from their household members. In addition, it deprives their household members especially the children or HIV infected contacts, who are more likely to develop disease and to present with severe forms of TB once infected, of their right and early opportunity to self-protection and access to TB services. Such TB services include; contact screening with initiation on preventive treatment, prompt TB diagnosis, and early initiation on appropriate TB treatment. This is an important reason why the “ethical and human right approach” has been proposed to guide the pursuit of END-TB targets [18].

The finding in our study that disclosure was motivated by the patient’s desire to attract care from household members further emphasize the culturally perceived role of family in Africa in caring for their patients. Such a perception though good, positions household members at risk of contracting TB as they care for their patients if appropriate TB infection control measures are not promoted and enhanced at this level. On the other-hand, it could be expected that anything that could deny a patient this care and support from household/family members could potentially impact on their psychosocial status, treatment adherence, and outcomes of treatment. Therefore, our findings further support the need for social and psychological support interventions within TB patient care [7].

Our study showed that being a female adult TB patient was associated with 6.5 times higher odds of partial or total non-disclosure as compared to males. In as much as the reasons for this gender disparity were not explored in this study, one potential explanation would be on the underlying fear of TB-related stigma and discrimination that could result from disclosure of TB status, just as it has been reported in studies conducted in Zambia and Ghana, with great effect on women than men [13, 19]. Other fears for disclosure among female TB patients could be on the risk of breaking marital harmony [14]. Importantly, this disparity raises important concerns with a need to explore gender issues within the efforts tailored towards TB control or elimination. Given that the study participants were from a setting in which women rely on their husbands for financial support to access healthcare, non-disclosure could deny women of this financial support for drug collection and could result in poor adherence as already reported in the context of patients on ART in Africa [20]. Also, given the culturally perceived role of women, that is, to care for people within their households, positions them as important agents of TB transmission especially to those dependent on their care like under-five children, their spouses and others in vulnerable states e.g. the HIV positive, pregnant women and elderly. More to this, given that the women in rural Africa, bare the greatest burden pertaining to household food production, children feeding, food preparation and child healthcare [21], issues related to their health could impact greatly on their children’s health, especially whether they could be enrolled on preventive treatment or evaluated for TB in the timely manner.

Belonging to the Muslim faith had 12.4 times higher odds of non-disclosure of TB status to household members as compared to patients belonging to the Catholic faith (p = 0.023). This is a new finding especially in the context of TB disclosure. However, shame-related HIV stigma has been reported to be strongly associated with religious beliefs such as the belief that HIV is a punishment from God or that people living with HIV/AIDS have not followed the Word of God [22]. It is however not clear whether such shame-related stigma, motivated by religious beliefs do exist in the context of TB, and if it could explain the disparity in religions with respect to disclosure to household members.

Although statistically non-significant, being single was associated with 4.6 times higher odds of non-disclosure of TB status to household members as compared to those who were married. This is a surprising finding that though may not be explainable based on the data available in this study, but will necessitate further exploration in not only a larger analytical study but also using qualitative methods.

As expected, our study reports positive rewards for majority of patients (>98%) following TB disclosure to their household members. The commonest reported positive rewards include; household members supporting their medication intake, offering encouragement, supporting their feeding, and financial support to enable attendance of clinic days. As expected, such positive rewards of disclosure, were considered by patients to have impacted on their treatment adherence and psychosocial aspects. This finding affirms the benefit of a successful engagement of family/household members in TB patient care through patients’ TB disclosure, a known foundational strategy in ensuring good treatment outcomes and infection control [11].

Surprisingly, all patients that had negative experiences following TB disclosure to household members also had atleast one positive experience concurrently. The reported negative experiences especially; marital separation, but also blame and negative criticism, and isolation, seem to indicate the existence of severe effects of potential underlying TB-related stigma among household members and communities at large. These findings are in agreement with those reported in previous studies on patients’ TB disclosure to household members, that reported isolation, divorce, among other outcomes [14]. Such negative post-disclosure experiences could be explained by the low awareness about TB disease that could be existing among patients and household members, something that could complicate TB control [23]. Such deficit in knowledge on TB by household members of TB patients, could be represented indirectly in this study by the fact that counseling on infection control and hope for total cure from TB, where the least performed aspects by household members following patients’ TB disclosure. Such deficit in TB knowledge, could offer alternative explanation on why negative experiences erupted concurrently with positive experiences of patients post disclosure. These findings, not only highlight the need for robust interventions aimed at enhancing social and psychological support to TB patients [7] through TB awareness, but also interventions that could directly promote TB disclosure to household members while minimizing on such negative outcomes.

Enhancing the involvement of treatment supporters in facilitating successful stigma-free disclosure of TB by patients to household members through training and provision of suitable TB information aides by healthcare workers, is one potential intervention that was perceived acceptable to two-thirds of TB patients in the study. Since the treatment supporter system is already in existence to support home-based DOTS, and that all TB patients are required to have one where necessary prior to treatment initiation, such disclosure enhancement interventions centered on treatment supporters could easily be adopted. However, the feasibility and efficacy of such models would need to be evaluated in future randomized trials, and its success would depend largely on the healthcare workers’ level of knowledge of TB, an argument supported by the already known positive correlation in knowledge on infection control practices between healthcare workers and household members [24]. Efficacy would also depend on the availability of acceptable TB information aides for use by both patient and treatment supporter, so as to facilitate the disclosure process to household members.

The study has several limitations: i) The small sample size may have reduced the power to detect significance of some predictor variables of non-disclosure. This may also limit the conclusions on the predictors of TB non-disclosure to household members ii) The study did not quantify the level of stigma among patients, in as much as it is known to influence disclosure. iii) Patients’ disclosure or not of TB to the household members was not assessed in regards to some clinical or treatment outcomes such as completion, cure, adherence or household contact screening, something that should be evaluated in future studies utilizing stronger analytical study designs. iv) The fact that the study never utilized qualitative assessment methods could not allow complete understanding of the major hindrances of disclosure especially among the female and moslem patients.

On the other-hand, our study unfolds the less addressed problem of TB patient non-disclosure to household members that could potentially impend the success of TB control strategies and attainment of the End TB targets of reducing TB incidence and TB-related death by 95% by 2035 [15]. Some of such TB control strategies hinged on successful disclosure to household members are: 1) TB Contact screening, which is impossible unless an index case voluntarily reports to have household contacts and willing to disclose to household members, 2) Initiation of TB contacts on preventive treatment, especially children, HIV infected adults, and other vulnerable household members, 3) Early TB diagnosis among symptomatic household members, which may be limited by delayed TB suspicion, hence delayed health seeking process by household members of TB patients, 4) Implementation of appropriate infection control measures at household level, 5) Home-based DOTS, and 6) Involvement of household members to offer social and psychological support to their patients, something that would impact on their treatment adherence and outcomes.

### Recommendations

Based on the study findings, we recommend that: 1) future larger studies should be conducted to conclusively establish the predictors of TB non-disclosure to household members, post-disclosure experiences, and their impact on treatment outcomes within both rural and urban settings; 2) Development of TB information aides to facilitate in process of patient disclosure to household members should be prioritized; 3) Interventions for enhancing disclosure at household level that are gender and religion-sensitive should be developed and evaluated in well-designed community-based randomized trials so as to ascertain their efficacy and impact on ensuring successful stigma-free TB disclosure, early access to TB diagnostic services, preventive therapy uptake, treatment adherence and extent of family support to the TB patients; 4) Psychosocial counselling should be integrated within the healthcare package for TB patients among TB burden countries; 5) Qualitative studies are needed to explore the reasons for non-disclosure among female and Muslim TB patients but also TB disclosure dynamics and experiences in general at a household level.

### Conclusion

The rate of non-or partial TB disclosure to household members is very high, with important differences across gender and religion. This poses a risk of missing an opportunity to control TB transmission at household level, access to available TB diagnostic, treatment and preventive services by household members, and attracting family support towards their patients’ care. Negative post-disclosure experiences reflecting severe forms of TB-related stigma, co-exist amidst the enormous patient-felt benefits of disclosure. There is need to enhance the knowledge of patients on TB infection control measures so as to minimize TB transmission at household level. Gender and religion sensitive interventions to enhance TB disclosure at household level while minimizing effects of stigma are urgently needed so as to increase engagement of household members in TB control efforts and patient care.

## Supporting information

S1 Dataset Minimal dataset. (CSV)

S1 Questionnaire English questionnaire. (DOCX)

S2 Questionnaire Runyankole questionnaire. (DOCX)
