# Supplementary material for: Non-disclosure of tuberculosis diagnosis by patients to their household members in south western Uganda
Source: PLoS One. 2020 Jan 24;15(1):e0216689. doi: 10.1371/journal.pone.0216689 (PMC6980409; doi:10.1371/journal.pone.0216689)
Supplement: S1 Questionnaire — (DOCX) [file pone.0216689.s002.docx]

**S1 Questionnaire. English Questionnaire**

**PART I: FOR PATIENT**

Study Number: |___|___|___|___|

|  | Patient Group  1= Intervention  2= Control | | | | | | | | | |  | | Date of baseline interview:  \|___\|___\|/\|___\|___\|/\|___\|___\|___\|___\| (dd/mm/yyyy) | | | | | | | | | | | | | |
| --- | --- | --- | --- | --- | --- | --- | --- | --- | --- | --- | --- | --- | --- | --- | --- | --- | --- | --- | --- | --- | --- | --- | --- | --- | --- | --- |
|  | Date of starting TB treatment  \|___\|___\|/\|___\|___\|/\|___\|___\|___\|___\| (dd/mm/yyyy) | | | | | | | | | | | | | | | | | | | | | | | | | |
| **A. SOCIODEMOGRAPHIC DATA** | | | | | | | | | | | | | | | | | | | | | | | | | | |
| 1 | | Gender  1= Male  2= Female | | | | | | | | | 2 | Date of TB diagnosis  \|___\|___\|/\|___\|___\|/\|___\|___\|___\|___\|  (dd/mm/yyyy) | | | | | | | | | | | | | | |
| 3 | What is your district of residence?  \|______________________________\| | | | | | | | | | | 4 | | What is your Parish of residence?  \|______________________________\| | | | | | | | | | | | | | |
| 5 | What is the residence type?  1= Rural [ ] 2 = Urban [ ] | | | | | | | | | | 6 | | What is your age?  \|___\|___\| Completed years | | | | | | | | | | | | | |
| 7 | What is your tribe?  1= Munyankore [ ] 2= Muganda [ ]  3= Mukiga [ ] 4= Others [ ] | | | | | | | | | | 8 | | If Other tribe, specify:  \|______________________________\| | | | | | | | | | | | | | |
| 9 | What is your level of education?  1=none [ ]  2=Primary education [ ]  3=Secondary education [ ]  4=Tertiary education [ ] | | | | | | | | | | 10 | | What is your marital status?:  1=Single [ ]  2= Married [ ]  3=Separated/divorced [ ]  4= Cohabiting [ ] | | | | | | | | | | | | | |
| 11 | What is your religious affiliation?  1=Catholic [ ] 2=protestant [ ]  3=Moslem [ ] 4=Pentecostal [ ]  5=Others [ ] | | | | | | | | | | 12 | | If other religion, specify:  \|___________________________\| | | | | | | | | | | | | | |
| 13 | What is your occupation?  1=Housewife [ ] 2= Business [ ]  3= Peasant Farmer [ ] 4= student [ ]  5= Civil servant [ ] 6=others | | | | | | | | | | 14 | | If Other occupation, specify:  \|______________________________\| | | | | | | | | | | | | | |
|  |  |  |  |  |  |  |  |  |  |  | 15 | | What is your income per month?  \|____________________________\| Ugsh | | | | | | | | | | | | | |
| **B. FAMILY RELATED FACTORS** | | | | | | | | | | | | | | | | | | | | | | | | | | |
| 16 | What family type do you belong to?  1 = Extended  2 = Nuclear  3= Single parent family  4 = Foster families | | | | | | | | | | 17 | | Number of Household members  \|____\|____\| | | | | | | | | | | | | | |
| 18 | Does any of your household members depend on you for upkeep?  1= Yes  2= No | | | | | | | | | | 19 | | Are you the head of the household?  1= Yes  2= No | | | | | | | | | | | | | |
| 20 | What is your relationship with the household head?  1= Not Applicable (self)  2= Spouse  3= Child  4= Brother/sister  5= Close relative  6= Other | | | | | | | | | | 21 | | If other type of relationship, specify?  \|_________________________________\| | | | | | | | | | | | | | |
| **C. BEHAVIOURAL FACTORS** | | | | | | | | | | | | | | | | | | | | | | | | | | |
| 22 | Where you taking alcohol just prior to TB diagnosis?  1 = Yes [ ] 2 = No [ ] | | | | | | | | | | 23 | | Are you currently taking alcohol?  1= Yes 2= No | | | | | | | | | | | | | |
| 24 | If stopped, when did you stop?  1= before starting TB treatment  2= During the first 2 weeks of treatment  3= After 2 weeks of treatment | | | | | | | | | | 25  a.  b.  c.  d.  e. | | Which type of alcohol? | | | | | | | | | | | | | |
|  |  |  |  |  |  |  |  |  |  |  |  |  | Type | | | | | | 1=Yes | | | | | 2=No | | |
|  |  |  |  |  |  |  |  |  |  |  |  |  | Waragi | | | | | |  | | | | |  | | |
| 26 | How many days a week were/are you taking alcohol? \|___\|___\| days | | | | | | | | | |  |  | Beer | | | | | |  | | | | |  | | |
|  |  |  |  |  |  |  |  |  |  |  |  |  | Spirits/wines | | | | | |  | | | | |  | | |
|  |  |  |  |  |  |  |  |  |  |  |  |  | Other Local brew | | | | | |  | | | | |  | | |
| 27 | Where were/are you taking alcohol from? | | | | | | | | | | 28 | | Do you smoke?  1 = Yes 2= No | | | | | | | | | | | | | |
|  |  | | | | 1=Yes | | | 2= No | | | 29 | | If stopped, when did you stop?  1= before starting TB treatment  2= During the first 2 weeks of treatment  3= After 2 weeks of treatment | | | | | | | | | | | | | |
|  | 1= At home | | | |  | | |  | | | 30 | | On average how many times were you smoking a day? \|____\|____\| times | | | | | | | | | | | | | |
|  | 2= In the bar | | | |  | | |  | | | 31 | | What do you commonly smoke?  1= Cigarettes 2= Tobbacco  3= Pipes 4= Malijuana | | | | | | | | | | | | | |
|  | 3=On Social functions | | | |  | | |  | | |  | |  | | | | | | | | | | | | | |
| **D. MEDICAL HISTORY** | | | | | | | | | | | | | | | | | | | | | | | | | | |
| 32 | Were you suffering from any of these conditions even before TB diagnosis | | | | | | | | | | 33 | | Are you on any treatment? | | | | | 34 | | | | How long have you been on treatment in months  ***(if applicable)*** | | | | |
|  |  | | 1=Yes | | | 2=No | | | 3=DK | |  | | 1=Yes | | | 2=No | |  | | | |  | | | | |
| a | Diabetes | |  | | |  | | |  | | a | |  | | |  | | a | | | | \|___\|___\|___\|___\| | | | | |
| b | HIV | |  | | |  | | |  | | b | |  | | |  | | b | | | | \|___\|___\|___\|___\| | | | | |
| c | Hypertension | |  | | |  | | |  | | c | |  | | |  | | c | | | | \|___\|___\|___\|___\| | | | | |
| d | Mental disorders (e.g depression) | |  | | |  | | |  | | d | |  | | |  | | d | | | | \|___\|___\|___\|___\| | | | | |
| e | Other, specify | | \|___________________\| | | | | | | | | d | |  | | |  | | d | | | | \|___\|___\|___\|___\| | | | | |
| 35 | Have you ever thought of taking your life (committing suicide) even before TB diagnosis? | | | | | | | | | | | | 1= Yes  2= No | | | | | | | | | | | | | |
| 36 | If on any treatment, which drugs is the patient taking per condition mentioned above? **(if applicable)** | | | | | | | | | | | | | | | | | | | | | | | | | |
|  |  | | | Drug 1 | | | | | | | Drug 2 | | | | | Drug 3 | | | | | | | Drug 4 | | | |
| a | Anti-diabetics | | |  | | | | | | |  | | | | |  | | | | | | |  | | | |
| b | ARVs | | |  | | | | | | |  | | | | |  | | | | | | |  | | | |
| c | Anti-hypertensives | | |  | | | | | | |  | | | | |  | | | | | | |  | | | |
| e | Mental illness | | |  | | | | | | |  | | | | |  | | | | | | |  | | | |
| d | Other, specify | | |  | | | | | | |  | | | | |  | | | | | | |  | | | |
|  |  | | |  | | | | | | |  | | | | |  | | | | | | |  | | | |
| 37 | Have you ever suffered from TB before this current episode?  1= Yes 2= No | | | | | | | | | | | | | | | | | | | | | | | | | |
| **E. TB DISCLOSURE FACTORS** | | | | | | | | | | | | | | | | | | | | | | | | | | |
| 38 | Have you disclosed your current TB status to your household members?  1= Yes, all  2= Yes, some  3= No | | | | | | | | | | | | | | | | | | | | | | | | | |
| 39 | If yes, what is your relationship with the household members you disclosed to? | | | | | |  | | |  | 40 | | | | If you disclosed, do you have a particular reason why you disclosed to the above people?  1= Yes 2= No | | | | | | | | | | | |
|  |  |  |  |  |  |  | 1=  Yes | | | 2= No | 41 | | | | If you have reasons, elaborate: | | | | | | | | | | | |
|  | 1= Father | | | | | |  | | |  |  |  |  |  |  |  |  |  |  |  |  |  |  |  |  |  |
|  | 2= Mother | | | | | |  | | |  |  |  |  |  |  |  |  |  |  |  |  |  |  |  |  |  |
|  | 2= Spouse | | | | | |  | | |  |  |  |  |  |  |  |  |  |  |  |  |  |  |  |  |  |
|  | 3= Child | | | | | |  | | |  |  |  |  |  |  |  |  |  |  |  |  |  |  |  |  |  |
|  | 4= Brother/sister | | | | | |  | | |  |  |  |  |  |  |  |  |  |  |  |  |  |  |  |  |  |
|  | 5= Close relative | | | | | |  | | |  |  |  |  |  |  |  |  |  |  |  |  |  |  |  |  |  |
|  | 6= Other | | | | | |  | | |  |  |  |  |  |  |  |  |  |  |  |  |  |  |  |  |  |
|  | If other, specify:  \|_________________________________\| | | | | | | | | | |  |  |  |  |  |  |  |  |  |  |  |  |  |  |  |  |
| 42 | If no disclosure yet, would you want to disclose to any of your household members?  1= Yes  2= No  3= Don’t know | | | | | | | | | | 43 | | | | If No, give reasons why you wouldn’t want to disclose.  \|________________________________\| | | | | | | | | | | | |
| 44 | If you desire to disclose, when would you want to disclose?  1= Immediately/as soon as possible (1-2days)  2= Later (3 or more days)  3= Not sure | | | | | | | | | | | | | | | | | | | | | | | | | |
|  | **POST-DISCLOSURE EXPERIENCES** | | | | | | | | | | | | | | | | | | | | | | | | | |
| 45 | **Can you tell us what your experience was two weeks after disclosing your TB status to your household members?**  ***(To be completed at baseline for Control group but on Follow-up for the Intervention group)*** | | | | | | | | | | | | | | | | | | | | | | | | | |
| a | **Positive experiences** | | | | | | | | | | **b** | | | | **Negative experiences** | | | | | | | | | | | |
|  |  | | | | | 1=Yes | | | 2=No | |  | | | |  | | | | | | 1=Yes | | | | | 2=No |
| i. | Received encouragement from family members | | | | |  | | |  | | i | | | | Criticism/blamed | | | | | |  | | | | |  |
| ii. | Support in taking my medications | | | | |  | | |  | | ii | | | | Isolated by household members | | | | | |  | | | | |  |
| iii | Support in feeding | | | | |  | | |  | | iii | | | | Withdrawn support | | | | | |  | | | | |  |
| iv | Received financial support to attend clinic days | | | | |  | | |  | | iv | | | | Marital separation | | | | | |  | | | | |  |
| v | Other | | | | |  | | |  | | v | | | | Other | | | | | |  | | | | |  |
| a.1 | If other, specify:  \|__________________________________\| | | | | | | | | | | b.1 | | | | If other, specify:  \|________________________________\| | | | | | | | | | | | |
| 46 | Can you tell us if any of the above experiences influenced on any of the following during the first 2 weeks on TB treatment? | | | | | | | | | | | | | | | | | | | | | | | | | |
| a |  | | | | | Positive experiences | | | | | | | | | b | | Negative experiences | | | | | | | | | |
|  |  | | | | | 1= Yes | | | | 2= No | 3=NA | | | |  | | 1= Yes | | | | | 2= No | | | | 3= NA |
| i | Treatment intake | | | | |  | | | |  |  | | | | i | |  | | | | |  | | | |  |
| ii | Proper Attendance of clinic visits | | | | |  | | | |  |  | | | | ii | |  | | | | |  | | | |  |
| iii | Self esteem | | | | |  | | | |  |  | | | | iii | |  | | | | |  | | | |  |
| iv | Hope of recovery | | | | |  | | | |  |  | | | | iv | |  | | | | |  | | | |  |
| v | Work | | | | |  | | | |  |  | | | | v | |  | | | | |  | | | |  |
| vi | Household harmony | | | | |  | | | |  |  | | | | vi | |  | | | | |  | | | |  |
| vii | Marital relationships | | | | |  | | | |  |  | | | | vii | |  | | | | |  | | | |  |
| 47 | Did you miss any dose of TB drugs during the first 2 weeks on treatment?  1= Yes 2 = No | | | | | | | | | | 48 | | | | How many days did you miss taking TB treatment during the first 2 weeks on treatment? \|___\|___\| | | | | | | | | | | | |
| 49 | If missed any dose, what were the reasons? | | | | | | | | | | | | | | | | | | | | | | | | | |
| 50 | Have you missed any scheduled visit during the first 2 weeks of TB treatment?  1= Yes 2 = No | | | | | | | | | | 51 | | | | If you missed any visit above, what were the reasons?  1= I forgot  2= Was discouraged/depressed  3= No transport  4= Was busy  5= Others  If other, specify: ……………………… | | | | | | | | | | | |
| 52 | Following your missed visit, after how many days did you come to the clinic? \|__\|__\| days | | | | | | | | | | 53 | | | | Did you have a treatment supporter at the time of starting TB treatment?  1= Yes 2= No | | | | | | | | | | | |
| 54 | What is your relationship with your treatment supporter?  \|________________________________\| | | | | | | | | | |  | | | | Are you still having the same treatment supporter that you had at the time of starting TB treatment?  1= Yes 2= No | | | | | | | | | | | |
| 55 | Which of the following has your treatment supporter been able to do for you? | | | | | | | | | | | | | | | | | | | | | | | | | |
|  |  | | | | | | | | | | | | | | | | | | | 1= Yes | | | | | 0= No | |
|  | Escorts me to the Clinic | | | | | | | | | | | | | | | | | | |  | | | | |  | |
|  | Picks my medications from clinic | | | | | | | | | | | | | | | | | | |  | | | | |  | |
|  | Reminds me of Clinic visit days | | | | | | | | | | | | | | | | | | |  | | | | |  | |
|  | Supervises me every when am taking medications at home | | | | | | | | | | | | | | | | | | |  | | | | |  | |
|  | Encourages me to complete my treatment | | | | | | | | | | | | | | | | | | |  | | | | |  | |
|  | Supports me financially | | | | | | | | | | | | | | | | | | |  | | | | |  | |
|  | Supports me with feeding | | | | | | | | | | | | | | | | | | |  | | | | |  | |
|  | Supports me in disclosing my TB status to other household members | | | | | | | | | | | | | | | | | | |  | | | | |  | |
|  | Supports me in passing information received at the health facility to household members | | | | | | | | | | | | | | | | | | |  | | | | |  | |
|  | Ensures that other household members especially children have less exposure with me to avoid infecting them? (e.g taking children away) | | | | | | | | | | | | | | | | | | |  | | | | |  | |
| 56 | If you were to disclose your TB status to other household members, would you mind if the healthcare worker trains your treatment supporter to assist in this disclosure? | | | | | | | | | | | | | | | | | | | 1=Yes 0=No | | | | | | |
| 57 | Do you think that giving information to household members on tuberculosis and how to manage and live with a patient with TB, could improve the support they give? | | | | | | | | | | | | | | | | | | | 0= No  1= Yes  2=Not sure | | | | | | |
| 58 | If Yes, in what would be your preferred channels for channeling this information to household members? | | | | | | | | | | | | | | | | | | | | | | | | | |
|  |  | | | | | | | | | | | | | 1= Yes | | | | | | 0= No | | | | | | |
|  | Written information with illustrative pictures | | | | | | | | | | | | |  | | | | | |  | | | | | | |
|  | Phone messages | | | | | | | | | | | | |  | | | | | |  | | | | | | |
|  | Phone call from Health worker | | | | | | | | | | | | |  | | | | | |  | | | | | | |
|  | Through treatment supporter | | | | | | | | | | | | |  | | | | | |  | | | | | | |
|  | Health worker visiting your home | | | | | | | | | | | | |  | | | | | |  | | | | | | |
|  | Video clips | | | | | | | | | | | | |  | | | | | |  | | | | | | |
|  | Through VHTs | | | | | | | | | | | | |  | | | | | |  | | | | | | |
|  | Yourself | | | | | | | | | | | | |  | | | | | |  | | | | | | |

THANK YOU FOR YOUR PARTICIPATION
