# Supplementary material for: Non-disclosure of tuberculosis diagnosis by patients to their household members in south western Uganda
Source: PLoS One. 2020 Jan 24;15(1):e0216689. doi: 10.1371/journal.pone.0216689 (PMC6980409; doi:10.1371/journal.pone.0216689)
Supplement: S2 Questionnaire — (DOCX) [file pone.0216689.s003.docx]

**S2 Questionnaire. Runyankole Questionnaire**

TITLE

Nibanyeta **Nyangoma Miria** amwegi wa Mbarara Yunivasite eya Sayansi na Tekinologiya ninshoma diguri y’eitembezo ry’akabiri omu bya Science Health Information Technology. Ninkora omushomo ogu ndikucondooza aha **by’omurwire w’akakonko akurabamu bwanyima y’abomukaye kuheebwa ekihandiiko ky’okushobora aha burwire bwa akakoonko yaba naraguriza ahirwariro rya akakoonko (TB Clinic) omwi Irwariro erikuru/ erihango erya Mbarara (Mbarara Regional Referral Hospital).**

Omushomo ogu nikimwe aha binshabirwe kukora nari kuhikiriza obwe naza kutunga diguri ye’itembezo rya kabiri omu Mbarara Yunivesity eya Sayansi na Tekinologiya.

Otorainwe kwejumbira omumushomo ogu nk’omwe ahab’omugasho omukugarukamu ebirakubuzibwe. Noshabwa kuba omwe ahabarayejumbe omumushomo ogu orikugarukamu ebirakubuzibwe ebiraije kumpweera omukucondooza

Ebigarukwamu nan’ebihandiko byaawe byoona nibaija kukwatwa nkekihama kandi nibyaija kukozesebwa ahaby’okweega kwangye kwonka hatari handi hoona. Noshabwa kugarukamu n’okuhereza ebitekateko byaawe nkoku orikwenda ahari ebyo byoona ebi orabuzibwe otagyemirwe. N’obu oyine emirimo mingi ninkushaba ompe obwiire bukye ongarukiremu ebibuuzo n’ebigarukwamu ebihikire nari ebishemereraire ekibuzo ekirabe nikikubuuzibwa . Eby’oragarukyemu nibisiimwa munonga.

Webaare munonga kwikiriza kukwatanisa naanye

EBIBUZO

ENAMBA Y’OMUSHOMO: |___|___|___|___|

| 1 | | Ekigombe ky’omurwaire  1= Abokuheebwa ekihandiiko  2= Abokubuzibwa kwonka | | | | | | | | 2 | | | Ebiro biba kuburizaho:  \|___\|___\|/\|___\|___\|/\|___\|___\|___\|___\| (ekiro/okwezi/omwaka) | | | | | | | | | | | |
| --- | --- | --- | --- | --- | --- | --- | --- | --- | --- | --- | --- | --- | --- | --- | --- | --- | --- | --- | --- | --- | --- | --- | --- | --- |
| A. SOCIODEMOGRAPHIC DATA | | | | | | | | | | | | | | | | | | | | | | | | |
| 3 | | | Ori omushaija nari omukazi)  1= Omushaija  2= Omukazi | | | | | | | 4 | | Ebiro ebibakukyebera bakakukwatamu akakoonko  \|___\|___\|/\|___\|___\|/\|___\|___\|___\|___\|  (ekiro/ okweezi /omwaka) | | | | | | | | | | | | |
| 5 | | Notuura omu disiturikiti eha?  \|______________________________\| | | | | | | | | 6 | | | Notuura omu Muruka guuha?  \|______________________________\| | | | | | | | | | | | |
| 7 | | Ahorikutuura no’mutowuni ninga omukyaaro?  1= omukyaaro [ ] 2 = Omutowuni [ ] | | | | | | | | 8 | | | Oyine emyaaka engahi?  \|___\|___\| | | | | | | | | | | | |
| 9 | | Oruganda rwawe niruuha  1= Munyankore [ ] 2= Muganda [ ]  3= Mukiga [ ] 4= Ezindi [ ] | | | | | | | |  | | | Waba otari wa rumwe aharuganda ezinagamba ngabira orwaawe  \|______________________________\| | | | | | | | | | | | |
| 10 | | Oshomire wahika nkaahi?  1=Tihariro [ ]  2=Omupuraimare [ ]  3=Omusiniya [ ]  4=Omubyemikono [ ] | | | | | | | | 11 | | | Washwire ninga washwirwe?  1=Ngaaha [ ]  2= Nshwirwe [ ]  3=Ntanire [ ]  4= Nintura n’omukundwa wangye [ ] | | | | | | | | | | | |
| 12 | | Noshoma diini ki?  1=Omukaturiki [ ] 2=Omuprotestanti [ ]  3=Omusilamu [ ] 4=Owamungu [ ]  5=Ezindi [ ] | | | | | | | |  | | | Waba noshoma ediini eyintagamba ngambira eyorikushoma  \|___________________________\| | | | | | | | | | | | |
| 13 | | Nokora murimo ki?  1=Ningumaomuka [ ]  2= omushubuzi [ ]  3= Omuhingi [ ] 4=Omwegi [ ]  5= Ninkora na’gavumenti [ ] 6= Ezindi [ ] | | | | | | | |  | | | Waba nokora ogundi murimo ogutari muryegi ngambira ogworikukora.  \|______________________________\| | | | | | | | | | | | |
|  |  |  |  |  |  |  |  |  |  | 14 | | | Notunga/nokora sente zingahi buri kwezi?  \|____________________\| Ush | | | | | | | | | | | |
| 15 | | Eka ey’orikurugamu nika ki?  1 = ey’abantu bingi [ ]  2 = ey’abantu bakye [ ]  3= ey’omuzeire omwe [ ]  4 = Mperekirwe yo [ ] | | | | | | | | 16 | | | Notuura nabantu bangahi omunjuyaawe/ahorikutuura?  \|____\|____\| | | | | | | | | | | | |
| 17 | | Okaba noonywa amaarwa ota kyebiieze akakoonko?  1 = Eego [ ] 2 = Apaana [ ] | | | | | | | | 18  a.  b.  c.  d.  e. | | | Okaba noonywa maarwa ki? | | | | | | | | | | | |
|  |  |  |  |  |  |  |  |  |  |  |  |  | Ekika | | | | | 1=Eego | | | | | 2=Apaana | |
|  |  |  |  |  |  |  |  |  |  |  |  |  | Waragi | | | | |  | | | | |  | |
| 19 | | How many days in a week were you taking alcohol? Korabe wabaire noganywa okaba oganywa emirundi engahi omusande? \|___\|___\| days | | | | | | | |  |  |  | Biya | | | | |  | | | | |  | |
|  |  |  |  |  |  |  |  |  |  |  |  |  | Ag’okusinza munonga | | | | |  | | | | |  | |
|  |  |  |  |  |  |  |  |  |  |  |  |  | Agandi agaburijo | | | | |  | | | | |  | |
| B. EBY’AMAGARA GAAWE | | | | | | | | | | | | | | | | | | | | | | | | |
| 20 | Orwire oburwaire obwobu; | | | | | | | | | | 21 | | | | Haine emibazi eyorikukoresa kutambira eburaire obwo? | | | | 22 | | Korabe nokoresa emibazi kitwire bwireki orikugikoresa? | | | |
|  |  | | | 1= Eego | | 2= Apaana | | | 3=Tinkumanya | |  | | | | 1= Eego | | 2=Apaana | |  | |  | | | |
| a | Shukaari | | |  | |  | | |  | | a | | | |  | |  | | a | | \|___\|___\|___\|___\| | | | |
| b | Akakookoka sirimu | | |  | |  | | |  | | b | | | |  | |  | | b | | \|___\|___\|___\|___\| | | | |
| c | Rwigazamitsi | | |  | |  | | |  | | c | | | |  | |  | | c | | \|___\|___\|___\|___\| | | | |
| d | Ebindi shoborora | | | \|___________________\| | | | | | | | d | | | |  | |  | | d | | \|___\|___\|___\|___\| | | | |
| 23 | Omurwaire yaba ari ahamibazi nimubazi ki ogwarikukoresa? | | | | | | | | | | | | | | | | | | | | | | | |
|  |  | | | | omubazi 1 | | | | | omubazi 2 | | | | | | omubazi 3 | | | | | | omubazi 4 | | |
| a | Ogwa Shukaari | | | |  | | | | |  | | | | | |  | | | | | |  | | |
| b | Ogwa sirimu | | | |  | | | | |  | | | | | |  | | | | | |  | | |
| c | Ogwa Rwigazamitsi | | | |  | | | | |  | | | | | |  | | | | | |  | | |
| d | Ogundi, shobora | | | |  | | | | |  | | | | | |  | | | | | |  | | |
|  |  | | | |  | | | | |  | | | | | |  | | | | | |  | | |
|  |  | | | |  | | | | |  | | | | | |  | | | | | |  | | |
| 24 | Aborikutura nabo wabagambire ku oshangirwe oyine akakonko?  1= Eego boona  2= Bamwe ni eego  3= Ngaaha | | | | | | | | | 25 | | | | Nokwatanisa ota naborikutura nabo abiwagambire korwaire akakoonko?  \|________________________________\| | | | | | | | | | | |
| 26 | Korabe wabagambire nahabwenki washazireho kubagambira  \|__________________________________________________________________________\| | | | | | | | | | | | | | | | | | | | | | | | |
| 27 | Korabe otaine owuwakigambireho notekateka kwija kukibagmbaho nabo?  1= Eego  2= Apaana  3= Tinkumanya | | | | | | | | | 28 | | | | Ekigarukwamu kyakuba kiri ngaaha nahabwenki otarikwenda bakimanye ngu orwaire akakoonko?  \|________________________________\| | | | | | | | | | | |
| 29 | Kuwakugira ekyetengo ky’okubagambira okakikora ryaari?  1= Naheeza kwihika omuka  2= Bwanyima  3= Tinkumanya | | | | | | | | | 30 | | | | Kuwakusharaho kubagambira nogira ngu okabagambira hahwaho bwire ki?  Ebiro: \|____\|_____\|  Esaande: \|____\|____\| | | | | | | | | | | |
| 31 | Nobaasa kutugambira ebiwarabiremu omu sande ibiri waheza kubagambaho nabeeka yaawe ku orwaire akakoonko | | | | | | | | | | | | | | | | | | | | | | | |
| a | Bakampwera kandi banyamba | | | | | | | | | b | | | | Bakanagijura kandi bakanyehara. | | | | | | | | | | |
|  |  | | | | | | 1= Eego | 2= Apaana | |  | | | |  | | | | | | 1= Eego | | | | 2= Apaana |
| i. | Nka kuhumurizibwa kunagambire abinkutuura naabo nyine akakoonko? | | | | | |  |  | | i. | | | | Bakancwera orubanja | | | | | |  | | | |  |
| ii. | Ba kampwera omukumira emibaazi yaangye | | | | | |  |  | | ii. | | | | Abinkutuura nabo bakanshorora ahabwokurwara akakoonko | | | | | |  | | | |  |
| iii | Ni bampwera omubyokurya | | | | | |  |  | | iii | | | | Obuhwezi obubabaire nibampa bakabunyihaho kubakimanyire ngu orwaire akakoonko | | | | | |  | | | |  |
| iv | Bakakuhwera omubya sente? | | | | | |  |  | | iv | | | | Omushaija waawe nari Omukazi waawe akataana naiwe ahabw’oburwaire? | | | | | |  | | | |  |
| v | Ebindi | | | | | |  |  | | v | | | | Ebindi | | | | | |  | | | |  |
|  | Haba hariho ebindi ebyakubaireho bigambe  \|__________________________________\| | | | | | | | | |  | | | | Haba hariho ebindi ebyakubaireho bigambe  \|__________________________________\| | | | | | | | | | | |

WEBARE / (MWEBAARE) MUNONGA KWEJUMBA OMUMUSHO OGU NOKUGARUKAMU GYE EBIBUO BYOONA.
